# Kinetic adaptation of human Myo19 for active mitochondrial transport to growing filopodia tips

**DOI:** 10.1038/s41598-017-11984-6

**Published:** 2017-09-14

**Authors:** Marko Ušaj, Arnon Henn

**Affiliations:** Faculty of Biology, Technion- Israel Institute of Technology, Haifa, 3200003 Israel

## Abstract

Myosins are actin-based molecular motors which are enzymatically adapted for their cellular functions such as transportation and membrane tethering. Human Myo19 affects mitochondrial motility, and promotes their localization to stress-induced filopodia. Therefore, studying Myo19 enzymology is essential to understand how this motor may facilitate mitochondrial motility. Towards this goal, we have purified Myo19 motor domain (Myo19-3IQ) from a human-cell expression system and utilized transient kinetics to study the Myo19-3IQ ATPase cycle. We found that Myo19-3IQ exhibits noticeable conformational changes (isomerization steps) preceding both ATP and ADP binding, which may contribute to nucleotide binding r﻿egulation. Notably, the ADP isomerization step and subsequent ADP release contribute significantly to the rate-limiting step of the Myo19-3IQ ATPase cycle. Both the slow ADP isomerization and ADP release prolong the time Myo19-3IQ spend in the strong actin binding state and hence contribute to its relatively high duty ratio. However, the predicted duty ratio is lower than required to support motility as a monomer. Therefore, it may be that several Myo19 motors are required to propel mitochondria movement on actin filaments efficiently. Finally, we provide a model explaining how Myo19 translocation may be regulated by the local ATP/ADP ratio, coupled to the mitochondria presence in the filopodia.

## Introduction

**Figure 1:**
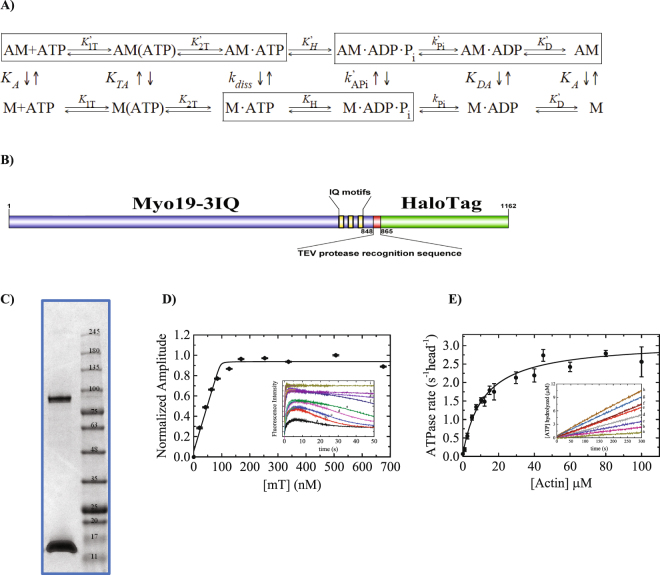
Myo19-3IQ expression, purification and its steady state ATPase activity. (**A**) The actomyosin ATPase cycle. The minimal reaction mechanism for the myosin’s conserved chemo mechanical energy transduction pathway, in boxes is the conserved predominate pathway, A-actin, M- Myosin, *K*′/*k*′ and *K*/*k* are the equilibrium/rate constants for the attached state (bound to actin) and detached states (unbound to actin), respectively. (**B**) Schematic representation of the expressed construct. (**C**) Coomassie Blue stained SDS-polyacrylamide gel of purified Myo19-3IQ heavy chain (97.3 kDa) (upper bands) and the associated calmodulin light chain (16.3 kDa) (lower bands). (**D**) The active-head titration measured by fluorescence increase of mT binding to myosin under single turnover conditions. The solid line through the data points is the best fit to Eq. 7 yielding active site concentration of 95 ± 6 nM. Error bars are within the symbols. *Inset*, time courses of mT binding by mixing constant amount of Myo19-3IQ and 15.63 (*a*) 31.25 (*b*), 46.88 (*c*), 62.5 (*d*), 93.75 (*e*), 125 (*f*) 187.5 (g), 250 (h) and 500 (i) nM mT. The solid lines to initial fluorescence increases are best fit to single exponential functions. (**E**) Myo19-3IQ steady-state ATPase activity as a function of [F-actin]. The solid line through the data points is the best fit to a rectangular hyperbola ($v={v}_{0+}({k}_{cat}[actin])/({K}_{m}+[actin]))$. Error bars represent standard deviation of at least three independent experiments from different protein purifications. *Inset*, time courses of ATP turnover with constant [Myo19-3IQ] and [Mg_2_ATP] at 1 (*a*) 3 (*b*), 7 (*c*), 11 (*d*), 21 (*e*), 60 (*f*), and 80 (*g*) and 100 (*h*) µM F-actin using the NADH-coupled assay.

Myosins are actin-based molecular motors that utilize energy from ATP binding, hydrolysis, and product release to do mechanical work^1^. These properties enable myosins to perform a plethora of cellular functions, such as muscle contraction, vesicle transport, cytoskeleton remodeling, tension maintenance, constriction of the cleavage furrow during cytokinesis, and more^2^. The myosin family comprises 35 different classes, defined based on amino acid sequence comparisons^3^. The primary structure of myosins includes a motor domain that binds actin and possesses the ATPase activity, a lever arm domain of one to six IQ motifs that bind diverse light chains, and a tail domain. The carboxyl-terminal domain is the most diverse part of myosins, exhibiting variance in length and in function, participating in protein–protein and membrane-binding interactions, among others^4, 5^. The motor domain is highly conserved and exhibits remarkable structural homology across all species and classes of myosin. Partial diversity in the motor domain can be seen in the variation of the surface loops such as loops 1 to 4, which contain various insertions or deletions, especially in loops 1 and 2^6, 7^. Despite the functional diversity of myosins, the actin-activated ATPase cycle and the mechanochemical transduction pathway are highly conserved across all myosins studied to date (Fig. 1A). However, the rates and the equilibrium constants of the internal biochemical transitions exhibit high heterogeneity across myosin classes and isoforms, resulting in unique enzymatic behavior for each myosin isoform in terms of cycling time, rate-limiting step, nucleotide-binding linkage, duty ratio (fraction of time that myosin spends strongly bound to actin), and force dependence of the biochemical transitions and intermediates^8, 9^. Furthermore, the specific enzymatic properties of each myosin define its ‘kinetic or enzymatic adaptation’ for its cellular function. Therefore, analysis of the detailed ATPase cycle is critical for understanding the myosin’s function.

Myosin 19 (Myo19), which is found only in higher vertebrates, was discovered as a novel mitochondria-localized myosin that affects mitochondrial motility when ectopically expressed^10^. Knockdown of Myo19 by RNA interference results in defects in mitochondrial segregation during cell division^11^. We recently reported a novel role for Myo19 in localization of mitochondria to the tips of filopodia in response to glucose starvation^12^. We and others have also identified a unique region within the myosin carboxyl-terminal domain that is both necessary and sufficient for mitochondrial targeting, which is mediated via tight interactions with the outer mitochondrial membrane^12, 13^. Myo19 is a barbed end motor with a sliding actin velocity of 230^14^ or ~50^15^ nm s^−1^ for the Myo19-3IQ construct, respectively. Based on ADP release rate constant Myo19 is suggested to be a high–duty ratio motor^15^. However, the key features of the Myo19 ATPase cycle remain to be elucidated, including its nucleotide-binding kinetics, thermodynamic coupling between the actin- and nucleotide-binding sites, and the nature of the biochemical transition(s) that account for the rate-limiting step. The identification of the unique enzymatic adaptation is essential for understanding how this motor is tailored for its cellular function^16, 17^.

In this work, we characterized the detailed enzymatic properties of Myo19-3IQ, which comprises the motor domain and the three IQ light chain–binding motifs. We expressed Myo19-3IQ in suspension-adapted, serum-free, human HEK293SF-3F6 cells, thereby achieving ideal conditions for proper protein folding and maturation. Using steady-state and transient kinetics, we revealed key features that are essential for its cellular function. Significantly, Myo19 possesses at least two transitions under no load that contribute to the rate-limiting step of its ATPase cycle. Our experimental dissection of its detailed enzymology, together with kinetic modeling, support the idea that Myo19 is a relatively high–duty ratio motor. Furthermore, we provide quantitative experimental data that may account for how Myo19 translocates mitochondria in response to the ATP/ADP ratio in growing filopodia. This sensitivity to the ATP/ADP ratio allows the motor to switch between its crosslinking activity and continuous translocation along the filopodia tips, tracking their elongation.

## Results

### Expression, purification, and steady-state ATPase activity of human Myo19

The primary structural organization of the Myo19-3IQ-HaloTag construct is shown in a schematic representation (Fig. 1B). This construct was co-expressed with calmodulin as its light chain in suspension-adapted, serum-free HEK293SF-3F6 human cells. The HaloTag is cleaved during elution to produce a motor domain with a lever arm of three IQ light chain–binding motifs that was termed Myo19-3IQ. Use of this expression system resulted in highly homogenous myosin that exhibited less than 3% variation in steady-state parameters among preparations. The key rate and equilibrium constants are presented in Table 1 and the complete list of all the rate and equilibrium constants are listed in Table S1. Quantifying the active enzyme concentration by active-site titration is critical for accurate determination of *k*_cat_, and has not yet been performed for this myosin isoform^14, 15^. Active-site titration for Myo19-3IQ was performed by single turnover measurements (STO) with mantATP (mT) (Fig. 1D)^18^ and fitted to Eq. 7 (Materials and Methods). The affinity for mT is reported in Table S1 (~*K*_1T_ 0.04 µM) which permitted this kind of assay. The steady-state parameters of Myo19-3IQ are shown in Fig. 1E and summarized in Table 1. Actin increased the steady-state ATPase activity of Myo19-3IQ by ~60-fold (*k*_cat_,_basel_ ≈ 0.05 s^−1^·head^−1^ to *k*_cat_ = 3.1 s^−1^·head^−1^), with a *K*_ATPase_ 11.6 ± 1.2 µM, which is the actin concentration at the half maximal ATPase activity (Table 1). The measured *k*_cat_ is in good agreement with the value obtained under similar conditions for human and mouse Myo19-3IQ expressed in Sf9 insect cells (Table S)^14, 15^. The *K*_ATPase_ for actin differs (~2-fold and ~3-fold tighter than reported for the human and mouse isoforms, respectively) from that in previous characterizations obtained under similar conditions (Table S1)^14, 15^. This may be due to differences in the expression system (insect *vs* human cells) and the different light chains used for the mouse Myo19-3IQ (LC9, LC12b listed Table S1)^15^. Next, we characterized the detailed enzymology by transient kinetics to reveal key biochemical intermediates of the ATPase cycle unique to Myo19-3IQ.

**Table 1:** Kinetic parameters for Myo19-3IQ.

*Parameter*	*Value*
	Myo19-3IQ
***Steady-state ATPase parameters***	
*k*_cat_ (s^−1^ head^−1^)	3.1 ± 0.1
*K*_ATPase_ (µM)	11.6 ± 1.2
*v*_o_ (s^−1^)	0.06 ± 0.02^5^
	0.08 ± 0.01^6^
***ATP binding***	
^*1*^ */K*′_1T_ (µM)	476 ± 116
*k*′_+2T_ (s^−1^)	1003 ± 26
*K*′_1T_ *k*′_+2T_ (μM^−1^s^−1^)^1^	2.17 ± 0.8
*k*′_−2T_ (s^−1^)﻿^﻿2﻿^	≈0
*k*′_+α_ (s^−1^)	17.5 ± 0.3
*k*′_−__α_ (s^−1^)^4^	2.6 ± 1.1
*K*′_α_^3^	6.8 ± 1.6
*K*′_0.5,slow_ (µM)	66
***ADP binding***	
*k*′_−1D_ (s^−1^)	9.3 ± 0.3
^*1*^ */K*_D,overall_ (µM)	0.13 ± 0.04

Conditions: 20 mM MOPS, pH 7.3, 50 mM KCl, 2 mM MgCl_2_, 0.2 mM EGTA, 1 mM DTT, 25 °C. ^1^Calculated parameter from rates or/and equilibrium constants. ^2^Calculated parameter from y-intercept. ^3^The equilibrium constant for isomerization, *K*′_*α*_, is define as *K*′_*α*_ = *k*′_+α_/*k*′_−α_. ^4^Calculated parameter as *k*′_−α_ = *k*′_*α*+_/*K*′_*α*_. ^5^NADH-coupled assay in the absence of actin. ^6^mT rate of fluorescence decay in the single turnover experiment.

### ATP binding to Acto·Myo19-3IQ

**Figure 2:**
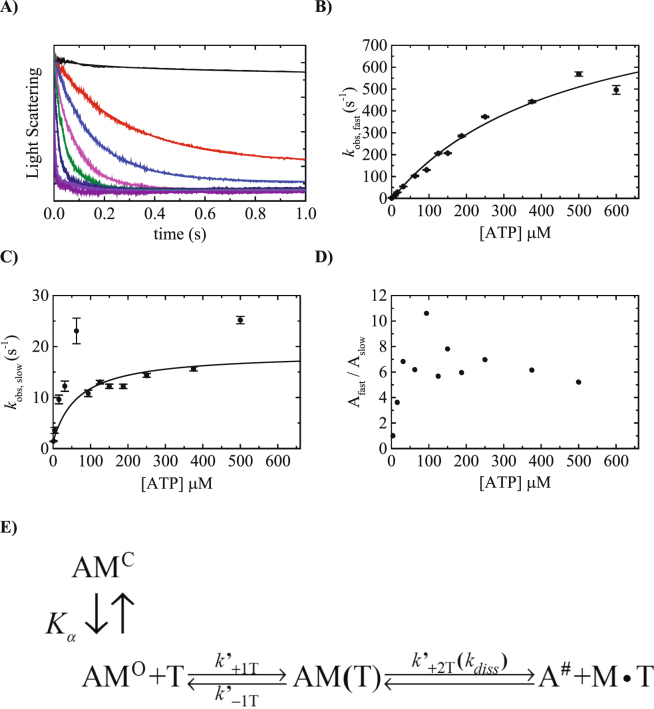
ATP binding to Acto·Myo19-3IQ. (**A**) Time course of light scattering decrease after mixing 0.25 µM Acto·Myo19-3IQ with increasing ATP concentration from 0 (upper trace) to 2, 4, 8, 16, 31, 63, 150 and 500 µM (bottom trace). Data are averaged transients (n = 3–5). The smooth lines through the data represent best fit to double exponential function. (**B**) [ATP]-dependence of the fast-observed rate constant of ATP binding to Acto·Myo19-3IQ as measured by light scattering. The straight lines through the data points are the best fits to Eq. 1. Error bars of the fitting are within data points. (**C**) [ATP]-dependence of the slow observed rate constants of ATP binding to Acto·Myo19-3IQ measured by light scattering. The straight lines through the data points are the best fits to hyperbola. Error bars of the fitting are within data points. (**D**) The ratio of the fast (A_fast_) to slow (A_slow_) amplitudes of the fluorescence transients as a function of [ATP]. **(E)** The kinetic reaction mechanism for ATP induced dissociation of Acto·Myo19-3IQ.

In contrast to many other characterized myosins, the binding of Myo19-3IQ to F-actin fluorescently labeled with pyrene, AEDANS, or coumarin did not result in any quenching or enhancement of fluorescence, limiting our ability to study its interaction with actin solely on the change in light scattering intensity. This suggests that the Myo19–actin binding interface differs in comparison with those of other myosins that are capable of quenching fluorescently labeled F-actin^19, 20^. Indeed, ATP binding to Acto·Myo19-3IQ induced dissociation of Myo19-3IQ from actin which can be seen by the change in light scattering (Fig. 2A). The dissociation of Acto·Myo19-3IQ upon ATP binding occurred with two phases that could be best fitted (see residual analysis Fig. S1) by a double-exponential function (Fig. 2A). Treating the Acto·Myo19-3IQ complex with apyrase for a prolonged period did not eliminate the slow phase upon mixing with ATP. The simplest kinetic mechanism that could account for this observation is one in which myosin has two nucleotide-binding conformations, with a slower equilibrium preceding a rapid nucleotide binding^21^. In one conformation, the nucleotide pocket is in the “open” state (AM^O^), which readily binds ATP and follows dissociation of actomyosin, indicated by the increase of light scattering. The second slower phase can reflect conformational changes (isomerization) preceding nucleotide binding. The pocket is in the “closed” state (AM^C^) that must isomerize to bind ATP. *k*_obs,slow_ describes the AM^C^ to AM^O^ interconversion, and *K*′_α_ is the isomerization equilibrium constant (kinetic scheme, Fig. 2E). The fast and slow observed rate constants (*k*_obs,fast_, *k*_obs,slow_) both exhibited hyperbolic dependence on [ATP] (Fig. 2B and C). Based on the kinetic scheme in Fig. 2E the fast-observed rate constant for ATP binding is predicted to follow a rectangular hyperbola and can be fitted according to Eq. 1:

1 $$k{\text{'}}_{obs,fast}=(\frac{K{\text{'}}_{1T}\cdot {k^{\prime} }_{+2T}[ATP]}{1+K{\text{'}}_{1T}[ATP]})+k{\text{'}}_{-2T}$$

The best fit of Myo19-3IQ to Eq. 1 yields an equilibrium constant (^1^/*K*′_1T_) for the initial rapid equilibrium ATP binding (*k*′_−1T_ >> *k*′_2T_, and *k*′_+1T_[ATP] + *k*′_−1T_ >> 1000 s^−1^) of 476 ± 116 µM and an isomerization rate constant (*k*′_+2T_ ≈ *k*′_diss_) to the dissociated state of 1003 ± 26 s^−1^. The intercept of the fit is indistinguishable from the origin; thus, ATP binding is essentially irreversible (*k*′_+2T_ >> *k*′_−2T_ ~0), consistent with the results of mT binding (Fig. 4A). The *k*_obs,fast_ hyperbolic dependence on [ATP] yields a second-order association rate constant for ATP binding for Acto·Myo19-3IQ, *K*′_1T_ *k*′_+2T_ = 2.17 ± 0.8 µM^−1^·s^−1^ (Table 1).

The observed slow phase during ATP-induced dissociation also exhibited hyperbolic dependence on [ATP] (Fig. 2C). The deviation from the model arises from the observed rate constants of the slow phase and the fast phase having comparable values, especially in the lower range of [ATP], i.e., *k*′_+α_ + *k*′_−α_ ~ *K*′_1T_ *k*′_+2T_ [ATP]^22^. The plot in Fig. 2C is shown as a “guide to the eye” to establish the maximum rate of the slow phase, *k*′_+α_ = 17.5 ± 3.3 s^−1^, and *K*_*0.5,slow*_ is 66 µM (Table 1)^23^. The ratio Amplitude_fast_/Amplitude_slow_ (A_fast_/A_slow_) reflects the distribution of AM^O^ and AM^C^ upon ATP binding (Fig. 2D), and thus defines the equilibrium *K*′_α_ = 6.8 ± 1.6. Knowledge of *K*′_α_ permits us to calculate *k*′_−α_ to be 2.6 ± 1.1 s^−1^. Thus, *k*′_+α +_ *k*′_−α_ = 20.1 s^−1^ and *K*′_1T_ *k*′_+2T_ [ATP] at 66 µM is 143 s^−1^, which is still observable and well separated from the slow phase and the ratio of the amplitude of both phases remain constant (Fig. 2D). This indicates that, although the AM^C^ state is inherited in the mechanism, the AM^O^ state is favorably populated.

### ADP binding to Acto·Myo19-3IQ

To study the unmodified ADP-binding kinetics of Myo19-3IQ, we used light scattering to monitor ATP-induced dissociation of actin from Acto·Myo19-3IQ·ADP complex^24^. Briefly, the rate of ADP release and the affinity of ADP for actomyosin can be determined by measuring the rate of ATP-induced dissociation of myosin from actin as a function of increasing levels of ADP prebound to actomyosin.

**Figure 3:**
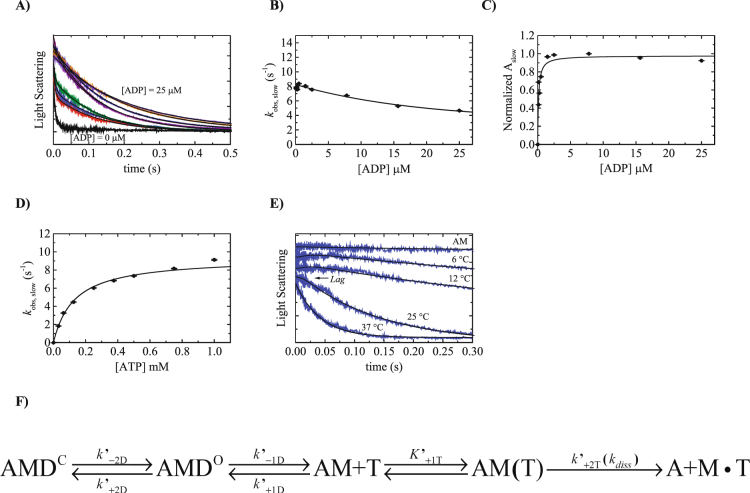
ADP binding to Acto·Myo19-3IQ as measured by kinetic competition with ATP. (**A**) Normalized time courses of light scattering decrease after mixing 0.2 µM Acto·Myo19-3IQ with 125 µM ATP in the absence or presence of different [ADP] ranging from 0.125 to 25 µM. Data are averaged transients (n = 3-5). Smooth lines through the data represent best fits to double or single exponential functions. (**B**) [ADP]-dependence of the slow observed rate constants for Acto·Myo19-3IQ as measured by kinetic competition with 125 µM ATP. (**C**) Normalized amplitudes of the slow phase obtained by fitting transients to double or single exponential functions as a function of [ADP]. The solid lines are fits of the data to Eq. 2. (**D**) [ATP]-dependence of the observed rate constants of Acto·Myo19-3IQ·D dissociation as measured by light scattering at saturated [ADP] of 25 µM. (**E**) The temperature dependence of the observed lag preceding ATP (500 µM)-induced Acto·Myo19-3IQ·D (0.2 µM) dissociation as measured by light scattering at saturated [ADP] of 25 µM. The solid line represents the best fit to the sum of exponential representing lag and the observed process as described previously^64^, error bars of the fitting are within data points. (**F**) The kinetic reaction mechanism for ADP release from Acto·Myo19-3IQ.

The time courses of Acto·Myo19-3IQ·ADP upon rapidly mixing with excess of ATP (125 µM), exhibited fast and slow phase or only slow phase at higher [ADP] (Fig. 3A). The observed fast phase rate constants report ATP binding to the fraction of ADP-free actomyosin, and their amplitudes decrease as [ADP] increases. At [ADP] ≥ 7 µM, the fast phase was no longer observed, and actomyosin dissociation exhibited only a single (slow) phase, which under these conditions, reflects the observed rate constant for ADP release (Fig. 3B). The kinetic scheme that follows this reaction mechanism is shown in Fig. 3F. Our analysis of ADP binding kinetics permitted us to extract several key kinetic parameters and their nomenclature is introduced in Fig. 3F and Table 1. Acto·Myo19-3IQ exhibited a slight dependence on [ADP], and the rate approached 3.3 ± 0.8 s^−1^, based on the fit (Fig. 3B). To obtain the affinity of ADP for Acto·Myo19-3IQ, we plotted the amplitudes of the slow phase (A_slow_) versus [ADP], as previously modeled (Fig. 3C)^25^. A_slow_ as a function of [ADP] exhibited a hyperbolic dependence and can be fitted according to Eq. 2:

2 $${A}_{slow}=\frac{[ADP]}{\frac{1}{{K^{\prime} }_{D}}+[ADP]}$$

which yields an overall very high affinity for ADP, (1/*K*_D_) = 0.13 ± 0.04 µM.

Myo19-3IQ has high affinity towards ADP and falls into the category of enzymes that need much higher [ATP] to determine the true ADP dissociation rate constant^24^. Therefore, we performed ATP-induced dissociation of Acto·Myo19-3IQ in the presence of saturating (25 µM) ADP with increasing [ATP]. Indeed, the *k*_obs_ of the single exponential transient (Fig. 3D) exhibited a hyperbolic dependence on [ATP], saturating at *k*′_−1D_ = 9.3 ± 0.3 s^−1^.

Interestingly, we observed a lag phase preceding the Acto·Myo19-3IQ dissociation (Fig. 3E) that became notable at high [ADP]. To substantiate the existence of this lag phase, we examined the temperature dependence of ADP release at high [ADP]. As the temperature decreased, we could clearly observe a longer lag phase (Fig. 3E). The existence of the lag phase suggests that ADP release occurs by sequential steps with comparable rate constants. This is consistent with the observation of the two fluorescent states, AMmD* and AMmD^#^, during mantADP (mD) binding (see below). Based on these features, a simple model for ADP release is described in Fig. 3F. Our results show that ADP binding and dissociation occur via multiple states that may contribute to the functional diversity of motor domain enzymology. Furthermore, ADP release is faster than the observed *k*_cat_ but within the range suitable to significantly contribute to the rate-limiting steps of Myo19 ATPase turnover.

### Kinetics of mantATP binding to Myo19-3IQ and Acto·Myo19-3IQ

**Figure 4:**
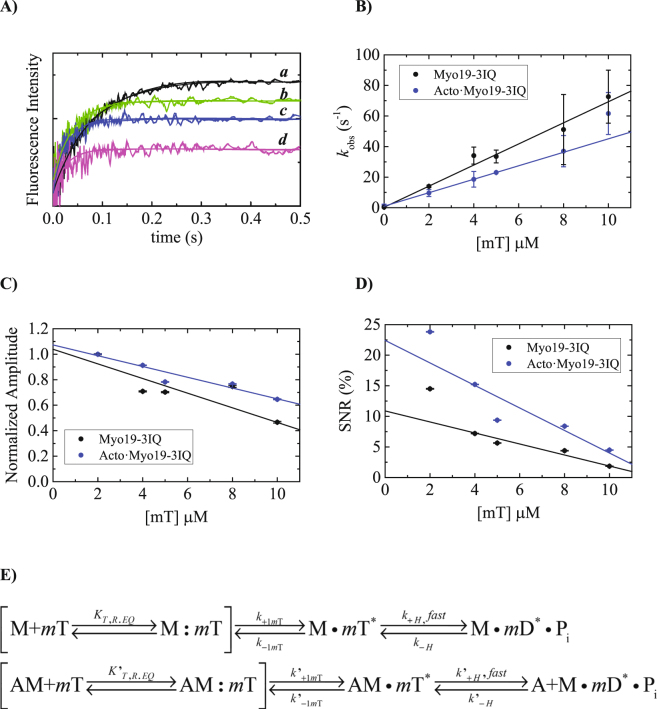
Binding kinetics of mT to Myo19-3IQ and Acto·Myo19-3IQ by monitoring FRET upon tryptophan excitation to mant-fluorophore. (**A**) Time courses of mT fluorescence change after mixing 200 nM myosin 19-3IQ with 2 (a), 4, (b), 5 (c), 10 µM (d) mT (final concentrations). The smooth lines are the best fit to a single exponential. (**B**) The dependence of *k*_obs_ on [mT]. The solid line through the data points is the best fits to linear equation. Error bars represent standard deviation from three independent experiments. (**C**) The dependence of the amplitudes on [mT]. The solid lines through the data points are the best fits to linear equation. Error bars of the fitting are within the data points. (**D**) The dependence of signal to noise ratio (SNR) of the experimental data on [mT]. Note the fast decrease in observable signals. (**F**) The kinetic reaction mechanism for mT binding to Myo19-3IQ and for Acto·Myo19-3IQ. We included in rectangular parenthesis a rapid collision complex that most likely occurs without fluorescence change.

Unlike many other myosins, Myo19-3IQ did not exhibit any increase in intrinsic fluorescence emission of aromatic residues upon ATP binding. Therefore, we monitored Förster Resonance Energy Transfer (FRET) between Myo19-3IQ (λ_ex, 280 nm_) and mant-labeled nucleotides upon binding (λ_em, >400 nm_). Time courses of fluorescence after mixing Myo19-3IQ or Acto·Myo19-3IQ with mT followed single exponentials (Fig. 4A) with observed rate constants (*k*_obs_) depending linearly on [mT], in the presence or absence of actin (Fig. 4B, Table S2). Interestingly, the amplitude of mT binding decreased as [mT] increased (Fig. 4C), although the observed rate constants increased linearly. Most likely the loss of amplitude can be attributed to the loss of fluorescence in the mixing or dead time of the instrument^26^. Alternatively, it was shown that a decrease in amplitude of the fluorescence transients in ATP binding experiments was observed with scallop heavy meromyosin (scHMM)^27^ and Dictyostelium myosin II^28^ with single tryptophan engineered at position 130. We show that a single tryptophan contributes to mT fluorescence (Shneyer *et al*. on Myo19^W140V^-3IQ mutant, parallel submission). To this end, both of these scenarios can contribute to the loss of amplitude. mT bound similarly to Myo19-3IQ or Acto·Myo19-3IQ, although the binding was faster in the absence of actin. Both y-axis intercepts are practically indistinguishable from the zero, suggesting that the high-fluorescence state of mT binding is irreversible. It is likely that a diffusion-limited collision complex in rapid equilibrium has spectroscopic properties similar to those of free Myo19-3IQ or Acto·Myo19-3IQ (Fig. 4F, square parenthesis), with *K*_T,R.EQ_, followed by a high-fluorescence state.

The kinetic model for ATP binding measured by light scattering shows additional isomerization described by *K*′_α_. In principle, this transition should also be observed upon mT binding to Acto·Myo19-3IQ. Despite the fact that we could potentially detect the *K*′_α_ transition at the lowest [mT], manifested either as a double-exponential process or as a lag preceding the fluorescence increase, these could no longer be reliably determined as [mT] increased. We conclude that the mT -binding assay cannot faithfully detect the *K*′_α_ transition, primarily due to the low signal-to-noise ratio (SNR) achieved in mT-binding experiments (see below), as in other myosin motors^29^.

The mT fluorescence signal, which arises from a single tryptophan, was relatively low for Myo19-3IQ (maximal fluorescence change ≈15% for myosin and ≈25% for actomyosin). To quantitatively estimate the net change, we calculated the SNR = (F0 − F∞/F0) × 100 (Fig. 4D), where F0 is the initial fluorescence of free mT and F∞ is the final fluorescence of free and bound mT. The resultant graph exhibits the same trend observed by plotting the amplitudes themselves, i.e., a sharp decrease in SNR as [mT] increases. It could well be that the weak signal is masked in the background fluorescence of the free mT. The effect was more pronounced for myosin than for actomyosin.

### Kinetics of mantADP binding to Acto·Myo19-3IQ reveal differences from its binding to Myo19-3IQ

**Figure 5:**
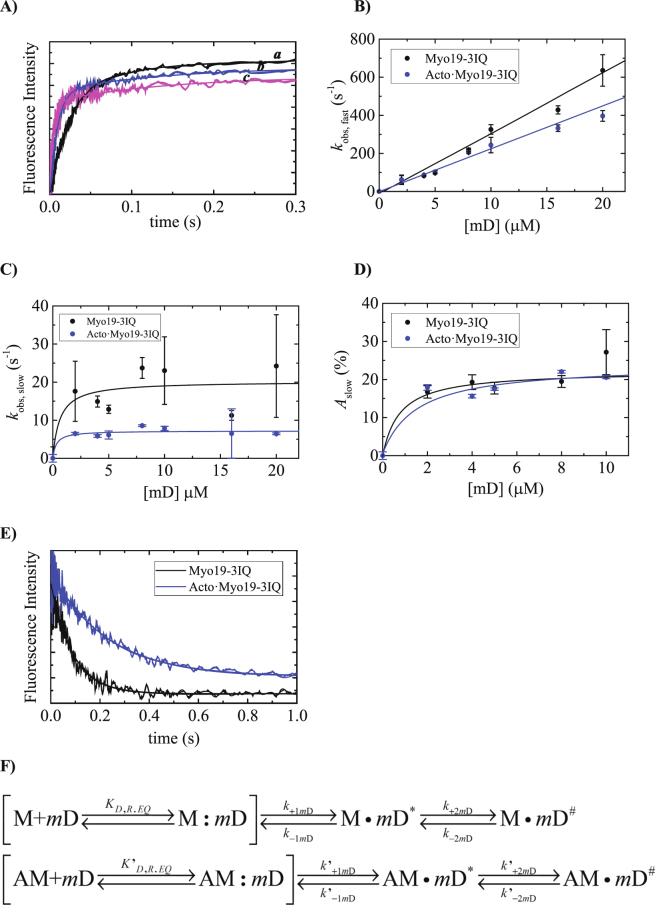
Binding kinetics of mD to Myo19-3IQ and Acto·Myo19-3IQ by monitoring FRET upon tryptophan excitation to mant-fluorophore. (**A**) Time courses of 2′/3′-mD fluorescence change after mixing 200 nM Myo19-3IQ with 2 (a), 5, (*b*) and 10 µM (*d*) 2′/3′-mD. The smooth lines are the best fit to a double exponential. (**B**) The dependence of *k*_obs, fast_ on [2′/3′-mD]. The solid line through the data points is the best fit linear function. Error bars represent standard deviation from two independent experiments. (**C**) The dependence of *k*_obs, slow_ on [2′/3′-mD]. The solid line through the data points is the best fit to hyperbola. Error bars represent standard deviation from two independent experiments. (**D**) The dependence of the amplitudes of the slow phase (*A*_slow_) on [2′/3′-mD]. The solid line through the data points is the best fit to hyperbola. Error bars represent standard deviation from two independent experiments. (**E**) The dissociation of the 2′/3′-mD from Myo19-3IQ and from Acto·Myo19-3IQ. The time courses of the 2′/3′-mD fluorescence change after mixing 250 nM Myo19-3IQ∙mD with 1000 µM ATP in the absence or presence of 275 nM phalloidin stabilized actin filaments. The smooth line is the best fit to single exponential function. (**F**) The kinetic reaction mechanism for mD binding to Myo19-3IQ and for Acto·Myo19-3IQ. In rectangular parenthesis, the rapid collision complex formation is shown most likely occurring without fluorescence change.

mD binding to Myo19-3IQ and Acto·Myo19-3IQ resulted in a fluorescence increase similar to that of mT (Fig. 5A), but without significant loss in amplitude as a function of increasing [mD]. Binding of mD to Myo19-3IQ and Acto·Myo19-3IQ followed double exponentials (Fig. 5B), with a fast-observed rate constant (*k*_mD,fast_) that depended linearly on the [mD] over the range examined and a slow observed rate constant (*k*_mD,slow_) that depended hyperbolically on [mD] (Fig. 5C). This kinetic behavior is more consistent with sequential binding mechanism for mD as follows. The fast phase reflects mD binding to the free Myo19-3IQ and Acto·Myo19-3IQ which results in the first high fluorescence state. The slower phase arises from isomerization of this initial high fluorescence state exhibiting hyperbolic dependence on mD. This is also in agreement with our observation of ADP binding kinetics as reported in Fig. 3F. A similar kinetic mechanism has been observed with mD binding to *mm*MyoVIIb-1IQ^30^, suggesting the existence of multiple ADP-bound states. Figure 5F provide the definition of the rate constants nomenclature utilized to describe mD binding to Myo19-3IQ and Acto·Myo19-3IQ, where the rate and equilibrium constants are provided in Table S2.

Under pseudo–first-order conditions ([mD] >> [Myo19-3IQ] or [Acto·Myo19-3IQ], and *k*_+1mD_·[Myo19-3IQ] or *k*′_+1mD_·[Acto·Myo19-3IQ] >> *k*_+2mD_ or *k*′_+2mD_), the observed rate constants of the fast phase reflected the formation of the first high-fluorescence state (Fig. 5B). It is likely that, in both reactions of mD binding to Myo19-3IQ and Acto·Myo19-3IQ, a spectroscopically silent complex is formed in rapid equilibrium before the formation of the first fluorescent state (Fig. 5F). This can be described by Eq. 3 ^30, 31^:

3 $${k}_{mD,fast}\approx {k}_{+1mD}[mD]-{k}_{-mD}$$

yielding comparable apparent second-order association rate constants, with *k*_+1mD_ for Myo19-3IQ of 31.8 ± 1.6 µM^−1^·s^−1^ and *k*′_+1mD_ for Acto·Myo19-3IQ of 22.5 ± 0.9 µM^−1^·s^−1^ (Table S2), based on the slopes. The intercepts, which define the dissociation rate constant of the first high-fluorescence state (*k*_−1mD_, *k*′_−1mD_), are indistinguishable from the origin, suggesting that the high-fluorescence state of mD binding is practically irreversible. The slow phase arose from isomerization of the initial high-fluorescence state to the second high-fluorescence state (Fig. 5C). The second slower phase exhibited weak dependence on [mD], and although it could be fitted to a hyperbola, it remained practically constant over the concentration range, with faster kinetics (~3 fold) for Myo19-3IQ than for Acto·Myo19-3IQ. This indicates that, at very low [mD], the maximal observed rate was reached. The observed rate constants of the slow phase are related to the rateconstants in Fig. 5C by Eq. 4 ^31^:

4 $${k}_{mD,slow}={k}_{+2mD}(\frac{{k}_{+1mD}[mD]}{{k}_{+1mD}[mD]+{k}_{-1mD}})+{k}_{-2mD}$$

which is the sum of the isomerization rate constants (*k*_mD,isom_ = *k*_+2mD_ + *k*_*−*2mD_) accounting for the population of the first high-fluorescence state that can undergo isomerization^30^. The maximal observed rate constant (*k*_+1mD_[mD] >> *k*_−1mD_ and *k*_+1mD_[mD]/*k*_−1mD_ >> 1) is equal to *k*_mD,isom_, and is more rapid for Myo19-3IQ (*k*_mD,isom_ = 20 ± 7 s^−1^) than for Acto·Myo19-3IQ (*k*′_mD,isom_ = 7.2 ± 1.2 s^−1^). The intercepts in Fig. 5C reflect the net dissociation rate constant (*k*_off_) of the second fluorescent state (Fig. 5F). However, they are not resolved in our case, and are indistinguishable from the origin because of poor data resolution at very low [mD]. This suggests that the second high-fluorescence state of mD binding is practically irreversible (similar to the first one). The amplitudes of the slow phase (Fig. 5D) exhibit similar weak dependence on [mD]. The fitted hyperbolas are not well defined, and yet the [mD] at half saturation predicts tight affinities of Myo19-3IQ and Acto·Myo19-3IQ for mD (<1 µM, Table S2).

mD dissociation was measured directly by competitive displacement of an equilibrated mixture of Myo19-3IQ·mD or Acto·Myo19-3IQ·mD with excess ATP (Fig. 5E). These values are in good agreement with a recently published study^15^, in which actin was shown to have a minor effect on ADP release. The dissociation of mD from either Myo19-3IQ·mD or Acto·Myo19-3IQ·mD competing with saturating ATP (1.0 mM) exhibited a single phase with a rate of 10.8 ± 1.2 s^−1^ or 4.6 ± 0.4 s^−1^, respectively (Fig. 5E, Table S2). Although we could clearly detect two mD binding relaxation transitions, the mD release exhibited a single phase. This is most likely due to the fact that the magnitudes of the dissociation rates were almost identical (see discussion of ADP dissociation measured by light scattering in ATP-induced dissociation of Acto·Myo19-3IQ·ADP complex), as both should be equally distributed under these conditions. Therefore, we consider *k*_off_ to be a reflection of the sum of the rate constants leading to loss of the first fluorescence state (*k*_off_ = *k*_−1mD_ + *k*_+2mD_) because, in our case, *k*_−1mD_ ≈ 0 and *k*_off_ ≈ *k*_+2mD_ for both Myo19-3IQ and Acto·Myo19-3IQ. Furthermore, because *k*_mD,isom_ = *k*_+2mD_ + *k*_*−*2mD_ (see above), *k*_*−*2mD_ ≈ 9.2 s^−1^ and *k*′_*−*2mD_ ≈ 2.6 s^−1^.

Due to the very high affinity of Acto·Myo19-3IQ for ADP and the limited data resolution, especially at lower mD, we could only obtain an approximation of the fitted kinetic parameters *k*_−1mD_, *k*_+2mD_, and *k*_*−*2mD_. This allowed us to calculate *K*_1D_, and *K*_2D_ and hence *K*_mD,overall_ using Eq. 5 (Table S2)^30^:

5 $${K}_{mD,overall}={K}_{1mD}(\frac{{K}_{2mD}}{1+{K}_{2mD}})$$

The overall affinity for mD is *K*_mD,overall_  ≪ 1 µM, but could not be accurately determined from the mD data alone (Table S2).

In summary, mD-binding kinetics were consistent with two well-defined, strong mD-binding states for Acto·Myo19-3IQ. Actin binding had little effect on the rate and equilibrium constants that govern mD binding and release from Myo19-3IQ, suggesting a weak coupling between ADP and actin binding.

### ATP hydrolysis of Myo19-3IQ and Actin-activated Pi release from Myo19-3IQ·ADP·Pi

**Figure 6:**
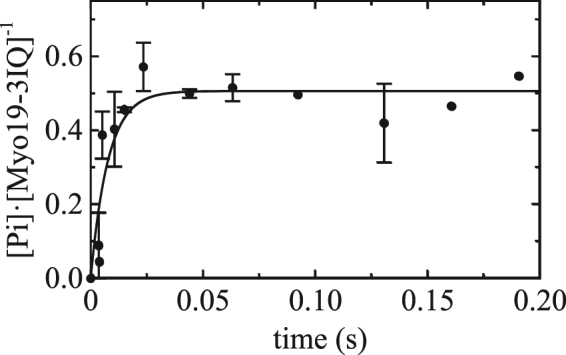
Time course of ATP hydrolysis and ADP·P_i_ burst of Myo19-3IQ. Time course of ADP-P_*i*_ formation by mixing 4 µM Myo19-3IQ construct with 250 µM. ATP (initial concentrations) followed by quenching with equal volume of 2 M HCl (premixing concentration). The solid line is the best fit of the data to the Eq. 10 with *k*_ss_ being slow and thus was ignored. Error bars represent standard deviation of 2-3 independent experiments.

Next, we directly measured the rate of ATP hydrolysis (*k*_+H_ + *k*_−H_) using a quenched-flow approach (Fig. 6). The ATP hydrolysis rate (*k*_+H_ + *k*_−H_) was ~129 s^−1^, much faster than the measured *k*_cat_ at saturating ATP. Myo19-3IQ (in the absence of actin) mixed with ATP yielded a P_*i*_ burst amplitude (*B*) of ~0.5 [P_*i*_]/[myosin]. The equilibrium constant for ATP hydrolysis in the absence of actin (*K*_H_), estimated from *B = K*_H_/(1 + *K*_H_), was ~1 (Fig. 6, Table S2).

**Figure 7:**
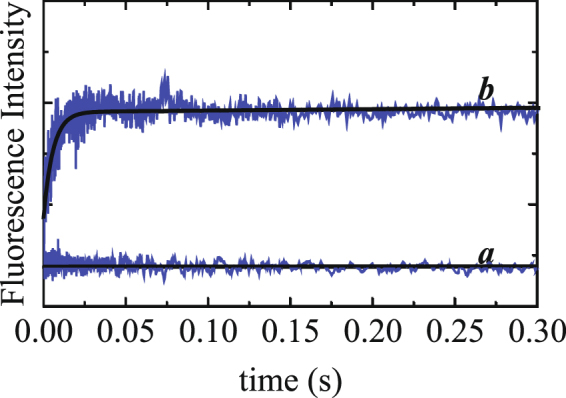
Actin-activated P_*i*_ release from Myo19-3IQ*·*ADP*·*P_*i*_. The time course of transient P_*i*_ release. Myo19-3IQ*·*ADP*·*P_*i*_ (0.5 µM) after mixing with 0 (**a**) or 80 µM (**b**) F-actin in the presence of 8 µM P_*i*_BiP. Myo19-3IQ*·*ATP were aged for 300 ms before mixing with F-actin (λ_ex_ = 436 nm, with 455-nm long-pass emission filter). The solid line represents the best fit to the sum of single exponential (initial burst) and linear (steady state turnover) function. Time course in the absence of the actin appears flat over the same timescale, as P_*i*_ release from myosin alone is very slow (0.05 s^−1^).

We then measured P_*i*_ release from Acto·Myo19-3IQ·ADP·P_*i*_ by performing double mixing experiments and monitoring P_*i*_ release upon binding of Myo19-3IQ·ADP·P_*i*_ to F-actin in the presence of P_*i*_BP (Fig. 7). The time course of P_*i*_ release after mixing Myo19-3IQ·ADP·P_*i*_ with 80 µM phalloidin stabilized F-actin (near saturating [actin] based on the *K*_ATPase_) showed a rapid exponential phase followed by a slow linear phase (Fig. 7). The burst corresponds to the first turnover of P_*i*_ release after actin binding, and the linear phase reflects steady-state ATP turnover. The observed rate of actin-activated P_*i*_ release (*k*′_−P*i*_) at 80 µM F-actin was 172 ± 6 s^−1^, indicating that P_*i*_ release does not limit the ATPase cycle in the presence of actin. The maximal rate for P_*i*_ release was not determined. There was no burst phase in the absence of actin (Fig. 7) because P_*i*_ release is rate-limiting (*k*′_−P*i*_ = 0.05 ± 0.2 s^−1^). In the presence of P_*i*_BP, P_*i*_ release is irreversible, and so the reverse P_*i*_-binding reactions (*k*_+P*i*_ and *k*′_+P*i*_) were not considered in the analysis.

### Implication of the kinetic mechanism on the duty ratio of Myo19-3IQ

**Figure 8:**
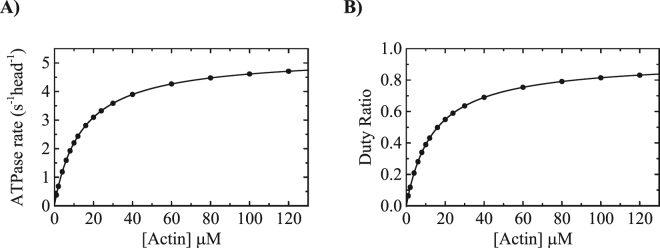
Simulation of the ATPase activity and duty ratio of Myo19-3IQ (**A**). Simulated steady state [actin]-dependence of the ATPase activity of the Myo19-3IQ. The solid lines through the data points are the best fit to a rectangular hyperbola ($v={v}_{0+}({k}_{cat}[actin])/({K}_{m}+[actin]))$. (**B**) Duty ratio as a function of [actin]. The duty ratio was calculated from the summation of all the biochemical intermediates distribution at each [actin] at the steady-state time regime according to equation: duty ratio = (strongly bound states)/(strongly bound states + weakly bound states). The solid lines through the data points are the best fit to a rectangular hyperbola ($duty\,ratio=1\,\ast \,[actin]/({K}_{DR}+[actin])$).

To model our ATPase cycle of Myo19-3IQ, we constructed a simulated steady-state curve by incorporating the measured and calculated equilibrium and rate constants into a whole ATPase cycle (Fig. 1A) utilizing KinTek Explorer^32^. Our simulation (Fig. 8A) yielded *K*_ATPase_ and *k*_cat_ of 14.1 ± 0.004 µM (experimentally determined as 11.6 ± 1.2 µM) and 5.2 ± 1.45 s^−1^ head^−1^ (experimentally determined as 3.1 ± 0.1 s^−1^ head^−1^). If we calculate the expected *k*_cat_ based on the ADP isomerization (17.5 s^−1^) and ADP release (9.5 s^−1^) being the predominant slower rates *k*_cat_ ≈ 6.1 s^−1^ head^−1^). Firstly, the *K*_ATPase_ we measured and reconstructed in the simulation are nearly the same. Secondly, the *k*_cat_ deviates by nearly 2-folds from the experimentally measured in this study and in others (Table S1). This may suggest that ADP isomerization and release during cycling may be slower than determined by ATP induced dissociation of ADP from the Acto·Myo19-3IQ·ADP experiments.

The *K*_DR_ (i.e., the [actin] at which 50% of the myosin heads are in a strong actin-binding state) is 13.8 ± 0.02 µM (Fig. 8B). Similarly, the duty ratio can be calculated according to De La Cruz *et al*.^33^, if ATP binding, hydrolysis, and P_*i*_ release are fast and ADP release limits the ATPase cycle using the following Equation 6:

6 $$Duty\,ratio=\,\frac{{k}_{+Api}[Actin](\frac{{K}_{H}}{1+{K}_{H}})}{{k}_{+Api}[Actin](\frac{{K}_{H}}{1+{K}_{H}})+{k}_{-D}}$$

which is true for Myo19-3IQ. Knowledge of *k*_APi_, *K*_H_, and *K*_−D_ (~1.0 µM ^−1^ s^−1^, 1.07 and 6.1 s^−1^, respectively), where *k*_APi_ is the lower limit estimation based on the measured *k*_Pi_, the *K*_DR_ is 12.0 ± 0.02 µM. If we assume the high actin filaments concentration in the filopodia, we can estimate that Myo19 to reach a duty ratio of ~0.8 under saturating [ATP] will require actin concentration ~50 µM. Similar to other myosins with relatively high duty ratios, Myo19 exhibits a high correlation between *K*_*ATPase*_ and *K*_DR_ because the Acto·Myo19·ADP is the dominant intermediate during steady-state cycling^17^.

## Discussion

In this study, we characterized the reaction mechanism for the actin-dependent ATPase cycle of human Myo19-3IQ using transient kinetics. Myo19-3IQ exhibits open (AM^O^) and closed (AM^C^) nucleotide states that are revealed upon ATP binding. Furthermore, Myo19-3IQ exhibits two ADP-binding states that both contribute to the rate-limiting step of the ATPase cycle. Myo19-3IQ has very high affinity towards ADP, with a weak coupling between Myo19-3IQ actin and ADP. We found that the *K*_DR_ for Myo19-3IQ was already high at low actin concentration, and is nearly identical to the *K*_ATPase_ affected by *k*′_APi_. Myo19 is predicted to be a monomeric motor, with moderate DR and cycling time. However, for a monomeric motor with a DR of <0.95 it may be required to work in an ensemble to form continuous movement^34^. In addition, our cell biology microscopy studies both by over expression and endogenous Myo19 suggest multi- motors are bound to a single mitochondrion^12^ (Shneyer *et al*., parallel submission). Finally, we suggest based on these results that Myo19 operate by an ensemble of motors bound to mitochondria.

### Analysis of ADP isomerization states in context with the ATP-binding model of Myo19-3IQ

This ATP-binding behavior is similar to what has been previously observed with class I myosins, including brush border Myosin-I^35^, Myo1b^36^, and Myo1c^37^. In the proposed mechanism, the myosin head is in equilibrium between two nucleotide-binding states, “open” (AM^O^) and “closed” (AM^C^), the latter being unable to bind nucleotides (Fig. 2E, vertical reaction)^22, 25, 38–40^. This isomerization is believed to represent a swing of the converter/IQ regions of the myosin motor domain^6^.

Our ATP-induced dissociation of Acto·Myo19-3IQ in the absence and presence of ADP exhibits a close correlation, with maximal values saturating at 17.5 ± 3.3 s^−1^ and 9.3 ± 0.3 s^−1^, respectively. The rates of ADP release and the slow phase of ATP-induced actomyosin dissociation (*k*′_α_) are also similar to each other for Myo1c^38^, leading to the suggestion that these rates report a transition between similar (or even identical) structural states. According to the model, similar to nucleotide-free actomyosin, Acto·Myo19-3IQ·ADP also has two states, AMD^C^ and AMD^O^, that may utilize the same structural transitions. A recent study challenged this idea by showing that, in contrast to Myo1c WT, these rates are not correlated in Myo1c N-terminal mutants, suggesting that *k*′_+α_ and *k*′_+αD_ could well represent two completely different structural transitions in the ATPase pathway^41^. In our analysis, we kept the original assumption, meaning that the closed-to-open structural transition of the nucleotide pocket reflects the same basic physical properties whether ADP is present or not.

**Figure 9:**
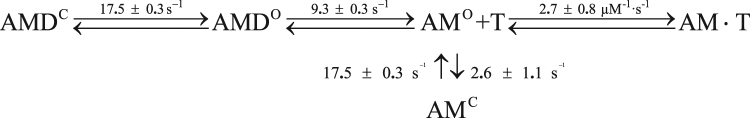
Kinetic reaction mechanism of the key transitions in Myo19 ATPase cycle contributing to the rate limiting step.

In principle, *k*′_+αD_ (ADP isomerization) should be extracted by AMD dissociation upon ATP binding by fitting transients to double exponentials. This is true when the two transitions possess quite different rate constants. Apparently, this is not the case for Myo19-3IQ, as the transients of ADP saturated Acto·Myo19-3IQ can be faithfully fitted only to a single exponential function. Therefore, we favor the following model for Myo19 ATP binding and ADP dissociation. In our model, we assume that AMD^C^ and AMD^O^ are in the same structural states as AM^C^ and AM^O^, and hence their transitions possess similar rates (i.e., *k*′_+α_ ≅ *k*′_+αD_). Secondly, the observed lag phase (Fig. 3E) in ADP dissociation experiments supports the existence of two sequential transitions with similar rates. In addition, two ADP states observed with mD binding could reflect the same transitions as described in Fig. 2E. Therefore, ADP release is a composite of two sequential transitions from AMD^C^ to AMD^O^, followed by AM^O^ with released ADP. Fig. 9 reaction mechanism summarizes these steps. Based on this mechanism, at least two transitions can contribute significantly to the Myo19 ATPase cycle under no external load. Using the rates in Fig. 9, the steady-state ATPase rate (*k*_cat_) is predicted to maximize at ~6.1 s^−1^ head^−1^, nearly twice as fast as our measured *k*_cat_ (3.1 s^−1^ head^−1^).

### Comparison with other filopodial myosins

Myo10 functions as a cargo transporter in intrafilopodial trafficking, filopodial growth and maintenance^17^. Interestingly, Myo10 has evolved structurally and kinetically to walk processively on parallel actin bundles by acquiring a unique dimerization domain that enables its mode of motility^42–44^. Although detailed kinetics studies of single headed Myo10^39, 43, 45, 46^ are different somewhat, it is clear that ADP release is faster than *k*_cat_ and does not contribute to the rate-limiting step. Better candidate for Myo10 rate limiting step seems to be the transition between the release of phosphate and the release of ADP which is slower than for other myosins and much closer to its *k*_cat_^47^ This transition has been compared between single headed Myo10 on actin filaments as oppose to a dimeric Myo10 on actin bundles^43^. The presence of actin bundles has increased by ~2-fold the rate of this transition and hence accelerating its motility^43^. It will be important to see if actin bundles affect in similar way Myo19 motility in filopodia. Collectively, Myo10 displays very different enzymatic properties tailored for different functions within the filopodia.

Myo7 from *Dictyostelium*, a structural and functional orthologue of Myo10, serves a function similar to that of Myo10 within filopodia^5, 48, 49^. On the other hand, Myo7 functions also as mechanotransductor where it generates and maintains tension. The main properties of Myo7 are a rate-limiting actomyosin ADP release, high affinities for ADP and F-actin, and a low degree of coupling between the actin- and nucleotide-binding sites^30, 50^. These specific kinetic adaptations indicate that Myo7 is a slow molecular motor with a high duty ratio, suitable for moving cargoes and mediating tension in the cytoskeleton^30, 50^. In this sense, (Fig. 4B and C) its kinetic mechanism is very similar to Myo19. It would be interesting to see if an artificial construct of Myo7 with mitochondria binding domain can translocate mitochondrion effectively to the filopodia tips.

Human Myo3A is thought to be monomeric, yet it traffics out to the tips of filopodia when overexpressed in cultured cells suggesting that it moves processively^51^. However, critical for Myo3A processive movement is a second, ATP-insensitive actin-binding site in the tail domain^52^. Myo3B, in contrast to its paralog Myo3A lacks a second actin-binding site in its tail domain. Myo3B can only target the tip of stereocilia when associated with its cargo protein espin-1 and other espin isoforms. Espin-1 is an actin-bundling protein that is proposed to increase the effective actin affinity of the transport complex, tether the Myo3B tail domain to the actin filament and limit its diffusion from actin^52^. This tethered motility establishes processivity and switches the nonprocessive Myo3B to a conditionally processive motor that “inchworms” to the tip of actin protrusions. The kinetic adaptation for this kind of processivity seems to involve slow rate of ATP binding whereas ADP release again follows a two-step mechanism^53^. Similar to Myo19-3IQ a transition between two actomyosin-ADP states is the rate-limiting step in the Myo3 ATPase cycle^53^. Moreover one of the actomyosin ADP state is particular long-lived with affinity for actin (*K*_d_ = 5 µM) being almost 40-times weaker than that of Myo19-3IQ^53^. Finally, none of the above filopodia motors, i.e., Myo7, Myo3, or Myo10, have been implicated in organelle transport to filopodia, further distinguishing Myo19^48, 52, 54^. However, for all of these myosins a kinetic adaptation signature is consistent with multiple actomyosin·ADP states.

### Implication of the coordinated translocation of Myo19 during filopodia elongation

**Figure 10:**
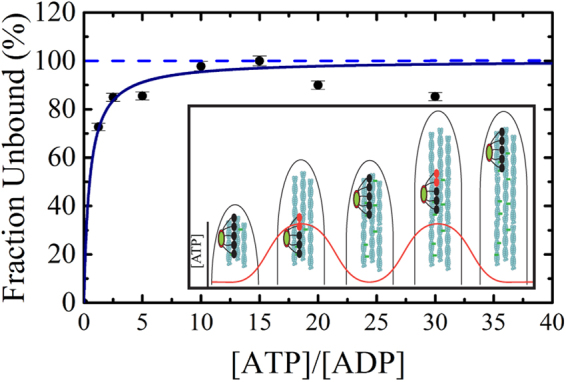
Working model for Myo19 active translocation within the filopodia. Fraction of unbound Acto·Myo19-3IQ·D (AMD) complexes as a function of ATP/ADP ratio. Fractions of unbound AMD complexes were calculated as the ratio between amplitudes observed with ATP induced AMD dissociation at saturated [ADP] = 25 µM and the total amplitude observed with ATP induced AM dissociation at no ADP present. Insert. Graphic representation of working model in the filopodia. Myo19 utilizes its ADP high affinity strong binding state to regulate its movement along the filopodia. Myo19 display ‘catch up’ movements with filopodia elongation and may fluctuate between stall and burst type movements as a function of the [ATP]/[ADP]. Actin polymerization and other cytoskeletal motors are dependent on the available ATP pool for their activity across the filopodia. ATP (and other small and large molecules) free diffusion is relatively slower due to fluid-phase viscosity in membrane-adjacent cytosol^65^. Hence, diffusion of ATP from the interior cytoplasm may not meet its local high demand inside filopodia. Motors are depicted as black circle (attached state) and red circles (detached states), mitochondria is colored in green. We acknowledge Boris I. Shneyer for his help in the making of the cartoon model in Fig. 10 inset.

In addition to ATP, which fuels motors described above, filopodia growth requires G-actin–ATP, which is critical for filopodia elongation by formins^55^. Currently, we propose that the mitochondria positioning by Myo19 is important for fulfilling the high ATP demand at growing filopodia tips. This may be reasoned by the limited ability of ATP to diffuse into the protrusion to fulfill the ATP demand. The assumption that the cytoplasm is a “well-mixed bag”, has been already challenged quite some time ago^56^. Furthermore, the mechanisms that can govern the relocation of mitochondria to the subcellular regions with high metabolic demands in neurons have been also proposed^57^. Thus, we integrated our results into a quantitative model that accounts for Myo19 enzymatic adaptation and filopodia growth (Fig. 10). The main pertinent feature of Myo19 with regard to translocation lies in its ability to switch from continuous runs to crosslinking activity, which we hypothesize depends on the local ATP/ADP ratio. Our experimental measurements (Fig. 10) show that, when the ratio of ATP/ADP increases to 5, only 20% of motor heads are in the strong actin-bound state(s). In that case, an ensemble of Myo19 motors on the mitochondrion could switch from crosslinking activity to processivity, because productive motility is more likely to occur in any given instance. On the other hand, when the ATP/ADP ratio decreases (i.e., due to high consumption of ATP at the elongating filopodia tips), the majority of motor heads would be in the strong actin-bound state(s), and the ensemble motility will be much less productive. When the ATP/ADP ratio increases locally due to production of ATP by bound mitochondria, the probability that the motor ensemble will produce motility increases. Due to a very high overall affinity towards ADP, we predict that Myo19 will be highly sensitive to changes in [ADP]. However, the presence of the mitochondria eventually increases the local [ATP] to the extent that it competes with the bound ADP (Acto·Myo19·ADP complex), enabling the growth of filopodia followed by Myo19 translocation. In future work, to formulate a quantitative model for Myo19-dependent transport, it will be essential to determine the motor stoichiometry or its density on the mitochondrion. It is likely that the physical properties of the mitochondrion together with myosin surface density regulate movement by a mechanical coupling mechanism^58^.

## Materials and Methods

### Reagents

All chemicals and reagents were of the highest purity commercially available. ATP was purchased from Roche Applied Science (Penzberg, Germany) and ADP was purchased from Bio Basic (Markham, ON, Canada). Nucleotide concentrations were determined by absorbance at 259 nm using ε_259_ of 15,400 M^−1^ cm^−1^. The N-methylanthraniloyl (mant) derivatives of the nucleotides, 3′-mant-2′-deoxy-ATP (mT), 2′/3′-mantADP (mD) were purchased from Jena Bioscience (Jena, Germany) and their concentrations was determined by absorbance at 259 nm using ε_255_ of 23,300 M^−1^ cm^−1^. In all experiments one molar equivalent of MgCl_2_ was added to nucleotide solutions immediately before use. N-(1-pyrene)iodoacetamide came from Molecular Probes (Eugene, OR, USA). 7-Diethylamino-3-[N-(2-maleimidoethyl)carbamoyl]coumarin, 3-(N-Morpholino)propanesulfonic acid (MOPS), Ethylene glycol-bis(2-aminoethylether)-N,N,N′,N′-tetraacetic acid (EGTA), Apyrase (potato grade VII), 7-Methylguanosine 5′-diphosphate sodium salt (7-MEG), purine-nucleoside phosphorylase (PNP, bacterial) and phalloidin were purchased from Sigma-Aldrich (St. Louis, Missouri, USA). Alternatively, the same form of phalloidin was purchased also from Setareh Biotech (Eugene, OR, USA). MgCl_2_∙6H_2_O came from Bio Basic (Markham, ON, Canada) and KCl from Merk (Darmstadt, Germany).

### Cloning of Myo19 cDNA constructs

The verified full-length human Myo19 cDNA^12^ was used as a template to amplify Myo19-3IQ construct with primers 3 and 4 (Table S3) carrying AsiSI and Pmel restriction sites. After restriction, the fragment was inserted into SgfI and Eco53kI cut pFC14K HaloTag CMV Flexi Vector (Promega, Madison, WI, USA). Myo19-3IQ-HaloTag fragment was inserted into pF12A RM Flexi Vector (Promega), upgraded in-house with puromycin resistance gene to enhanced generation of stable cell lines according to manufacture protocol (Promega). pF12A RM Myo19-3IQ-HaloTag was generated by restriction ligation using AsiSI and SbfI where pFC14K Myo19-3IQ was a donor vector and pF12A RM was acceptor vector. The resulting plasmid (pF12A RM Myo19-3IQ-HaloTag) was then fully verified by sequence analysis. Human calmodulin light chain gene^10^ was cloned to pF4A CMV Flexi Vector (Promega) according to the manufacturer’s protocol using primer 1 and 2 (Table S3). All PCR reaction were done with Phusion High fidelity DNA polymerase (Thermo Fisher Scientific, Waltham, MA, USA) and T4 PNK and ligase (NEB, Ipswich, MA, USA) were used according to the manufacturer’s instructions.

### Expression and purification of Myo19 constructs

Detailed description of protein expression and purification will be published in separate method paper (under preparation). Briefly, suspension adapted HEK293SF-3F6 cells^59^ (obtained from ATCC) were grown in serum free media EX-CELL293 (Sigma-Aldrich) or in house made proprietary cell culture media. A pool of cells stably expressing Myo19-3IQ construct were used for protein expression and purification for the kinetic characterization. Promega regulated mammalian expression system was used according to the manufacturer’s instructions. Suspension cultures of HEK293SF-3F6 cells (typically 1-1.5 L at once) were transfected or induced for the expression of the desired Myo19 construct. The transfected or induced cells (initial concentration of 1-2 × 10^6^ cells/ml) were allowed to grow for 2-3 days at 37 °C while shaking (reaching 2–4 × 10^6^ cells/ml) and were then pelleted at 350 × g for 10 min. All protein purification steps were carried out on 4 °C. On the day of purification cell pellets were re-suspended in HaloTag system compatible lysis buffer (20 mM MOPS, 130 mM KCl, 5 mM MgCl_2_, 2 mM EGTA, 2 mM ATP, 0.5 mM DTT, 5% Glycerol, 0.05% NP-40, 2 µM calmodulin, 1 X Promega protease inhibitor cocktail, pH 7.5) to achieve cell density ≈ 40 × 10^6^ cells/ml and lysed by homogenization in a Dounce homogenizer with a glass pestle by ≈ 60 strokes. Lysates were supplemented with additional 1 mM MgATP and cleared by ultra-centrifugation at 100,000 × g for 1 h (4 °C). From this point on, the purification followed HaloTag purification system manuals and protocols provided by Promega. Final elution was concentrated using Millipore (Merck, Darmstadt, Germany) 3 kDa amicon ultra centrifugal filter unit and dialyzed into storage buffer (20 mM MOPS, 75 mM KCl, 5 mM MgCl_2_, 2 mM EGTA, 0.5 mM DTT, 50% Glycerol, pH 7.5). The purified Myo19-3IQ construct was stored at −20 °C. Myo19-3IQ construct concentration was determined using predicted extinction coefficient at 280 nm (λ_ex.coff, WT_ = 105,770 M^−1^ cm^−1^, ExPASy ProtParam). The proteins were re-suspended in 6 M guanidine hydrochloride (Sigma-Aldrich) and the absorption spectra was obtained in 40 µl ultra-micro quarts cuvettes by T90 + spectrometer (PG instruments, Leicestershire, UK) controlled by company software UWin. Subsequently the active heads concentration was determined by mT titration under single turnover conditions^18^. The obtained amplitudes of mT fluorescence increase were normalized and fitted according to Equation 7:

7 $$[Myo\cdot mT]=\frac{(R+n+\frac{{K}_{D}}{Myo})+\sqrt{{(R+n+\frac{{K}_{D}}{Myo})}^{2}-4\cdot R\cdot n}}{2\cdot n}$$

where Myo is the concentration of Myo19-3IQ determined by extinction coefficient, R is (experimental) ratio of [mT]/[Myo] or [mT] alone, [Myo∙mT] are normalized amplitudes, *K*_D_ is affinity of Myo19-3IQ for mT (set as 50 nM) and *n* is fitted value reporting concentration of active sites (or the true ratio of [mT]/[Myo]).

### Expression and purification of other proteins

Actin was purified from rabbit or chicken skeletal muscle labeled with pyrene if needed, and gel-filtered over Sephacryl S-300 HR^60^. Ca^2+^-actin monomers were converted to Mg^2+^- actin monomers with 0.2 mM EGTA and 40 µM MgCl_2_ (excess over [actin]) immediately prior to polymerization by dialysis against KMg50 buffer (20 mM MOPS, 50 mM KCl, 2 mM MgCl_2_, 0.2 mM EGTA, 1 mM DTT, pH 7.3 at 25 °C). Phalloidin (1:1 molar ratio) was used to stabilize actin filaments. 7-Diethylamino-3-((((2-maleimidyl) ethyl)amino)carbonyl)coumarin-labeled phosphate-binding protein (P_*i*_BP) was expressed, purified, and labeled as described^61^. Calmodulin was expressed and purified in bacteria as described^62^.

### Steady-state ATPase activity

Steady-state ATPase Activity - The actin-activated steady-state ATPase activity of Myo19-3IQ was measured at 25 ± 0.1 °C in KMg50 buffer supplemented with 2 mM MgATP using the NADH coupled assay^24^ by monitoring changes in absorption at 340 nm. The Myo19-3IQ concentration was 10-50 nM but constant during each individual experiment. Time courses of single turnover ATPase activity in the absence of actin were measured using ATP and mT.

### Stopped-flow measurements

All experiments were performed in KMg50 buffer with the Hi-Tech Scientific SF-61DX2 stopped-flow apparatus (TgK Scientific Limited, Bradford-on-Avon, UK) at 25 ± 0.1 °C. The concentrations stated through all the text are always final concentrations after mixing (i.e. in the observation cell) unless noted otherwise. Intrinsic tryptophan fluorescence (λ_ex_ = 280) was measured through a Schott 320WG filter. Light scattering was measured at 90 ° with excitation at 313 nm. Most time courses shown are of individual, 2000-point transients collected with the instrument in oversampling mode, where the intrinsic time constant for data acquisition is ≈64 µs. Typically, multiple (3 to 5) time courses were averaged before analysis. Time courses displayed fast and slow phases were collected on a logarithmic or split time scale. Using Kinetic Studio software provided with the instrument or with Origin (OriginLab Corporation, Northampton, Massachusetts, USA), time courses of signal (fluorescence, light scattering) change were fitted to a sum of exponentials according to Equation 8:

8 $$F(t)={F}_{\infty }+\sum _{i=1}^{n}{A}_{i}{e}^{-{k}_{i}t}$$

where *F*(*t*) is the signal at time *t*, F_∞_ is the final signal value, *A*_*i*_ is the amplitude, *k*_*i*_ is the observed rate constant characterizing the i-th relaxation process, and *n* is the total number of observed relaxations. The value of n was either one (single exponential) or two (double exponential). For mant-nucleotides binding, photobleaching of the fluorescence signal was not of any significance to affect fitting of the time courses within the 5 s. The dead time of the instrument determined from the reduction of 2,6-dichlorophenolindophenol with ascorbic acid in absorbance mode was 1 ms. Fitting was limited to data beyond 1 ms to account for the instrument dead time and to exclude data acquired during the continuous flow phase of mixing, as recommended by the manufacturer.

Uncertainties are reported as standard errors in the fits unless stated otherwise and were propagated using the general formula according to Equation 9:

9 $$da=\sqrt{{(\frac{\partial a}{\partial {x}_{1}}d{x}_{1})}^{2}+\mathrm{..}.+{(\frac{\partial a}{\partial {x}_{n}}d{x}_{n})}^{2}}$$

where the experimental measurements x_1_, x_2_… x_n_ have uncertainties dx_1_, dx_2_… dx_n_ and a is a function of x_1_, x_2_… x_n_.

### Nucleotide binding kinetics

Time courses of nucleotide binding were acquired under pseudo first-order conditions with [nucleotide] >> [myosin or actomyosin]. Actomyosin samples were prepared by mixing equal molar amount of Myo19-3IQ with actin filaments or, where specified, with [actin] >> [myosin]. Myosin and actomyosin samples were treated with apyrase (0.01 unit/ml) and equilibrated on ice for 10 min before measurements. The final apyrase concentration after mixing was 0.005 unit/ml used to deplete ATP and ADP from Myo19-3IQ and Acto∙Myo19-3IQ^24^.

### Transient phosphate release

Transient P_*i*_ release was measured using the fluorescently labeled mutant^61^ of the P_*i*_ binding protein (P_*i*_BP) with the instrument in sequential mixing mode^24, 30^. Myo19-3IQ (0.5 µM final concentration) was mixed with ATP under multiple turnover conditions (125 µM ATP final concentration) for 300 ms to allow ATP binding and hydrolysis to occur, and then rapidly mixed with saturating F-actin. P_*i*_BiP (8 µM) was included in the myosin, nucleotide and actin solutions. Background P_*i*_ was removed from all solutions, syringes and the instrument by incubating them with 7-methylguanosine (0.2 mM) and purine nucleoside phosphorylase (0.1 units mL^−1^). The obtained transients in the presence of actin were fitted as single exponential with a slope (steady-state) under multiple turnover conditions.

### Equilibrium constant for ATP hydrolysis

We measured the equilibrium constant for ATP hydrolysis in the absence of actin using Hi-Tech Scientific RQF-63 rapid quench-flow apparatus (TgK Scientific Limited, Bradford-on-Avon, UK). Measurements were done under multiple (250 µM ATP and 4 µM Myo19-3IQ, initial concentration) and single (3 µM ATP and 4 µM Myo19-3IQ, initial concentrations) turnover conditions. The reaction was terminated after ~3.9 ms −200 ms with quench solution (2 N HCl). The P_*i*_ of hydrolyzed ATP was directly determined using Colorimetric Phosphate Assay kit (Abcam, Cambridge, UK) according to manufacturer manuals. The phosphate concentration [P_*i*_] was normalized by dividing by the myosin concentration [Myo19-3IQ] and the time course was fitted by according to Equation 10:

10 $$\frac{[{P}_{i}]}{[Myo19-3IQ]}=B(1-{e}^{-{k}_{obs}t})+{k}_{ss}t$$

where *k*_obs_ = (*k*_+H_ − *k*_−H_) and *k*_ss_ is the steady-state turnover rate. The burst amplitude (*B*) is given by B = *K*_H_/(1 + *K*_H_). The burst amplitude (*B*) was additionally confirmed separately by manual mixing of Myo19-3IQ and ATP. The reaction was terminated after different intervals between 15–60 s with quench solution (2 N HCl), and liberated P_*i*_ was detected as above.

### Kinetic simulations and modeling

Kinetic simulations and modeling of reaction time courses were performed using KinTek Explorer^32, 63^. Simulations of the steady-state reaction time courses were performed according to the reaction mechanism shown in Scheme 1, 2 & 4 and the rate constants in Table 1 and Table S2.

### Data availability

The datasets generated during and/or analyzed during the current study are available from the corresponding author on reasonable request.

## Electronic supplementary material

Supplementary Information
