# Supplementary material for: Kinetic adaptation of human Myo19 for active mitochondrial transport to growing filopodia tips
Source: Sci Rep. 2017 Sep 14;7:11596. doi: 10.1038/s41598-017-11984-6 (PMC5599584; doi:10.1038/s41598-017-11984-6)
Supplement: Supplementary file 1 — Supplementary Information [file 41598_2017_11984_MOESM1_ESM.pdf]

Kinetic adaptation of human Myo19 for active mitochondrial transport to growing filopodia tip

Supplementary Information

Authors: Marko Ušaj and Arnon Henn*

Faculty of Biology, Technion- Israel Institute of Technology, Haifa, 3200003, Israel

*Address correspondence to: Arnon Henn, Faculty of Biology, Technion- Israel Institute of Technology, Haifa, 3200003, Israel Tel. +947 (4) 8294839; Fax. +947 (4) 8295424; email: arnon.henn@technion.ac.il

Figure S1: Residual analysis for single and double exponential fitting for ATP induced dissociation of Acto·Myo19-3IQ by light scattering

A)

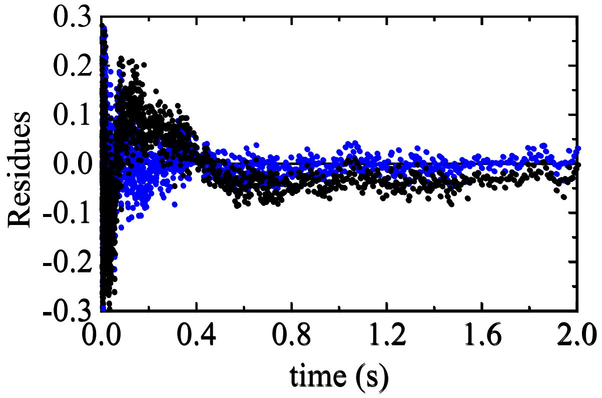

B)

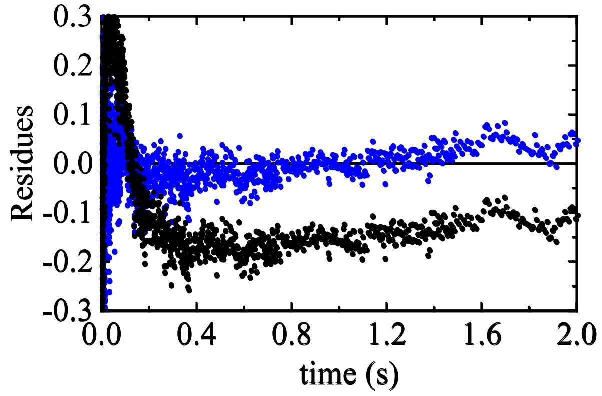

C)

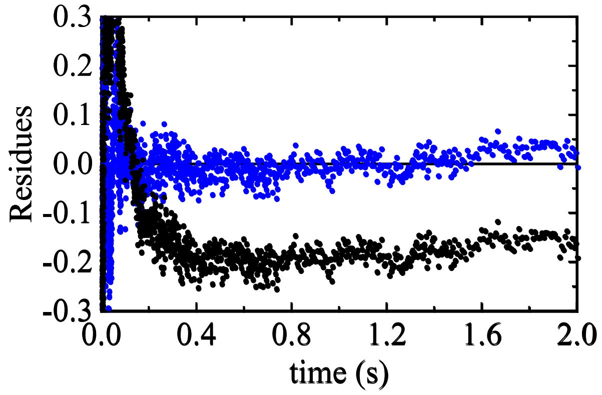

Figure S1: **Residual analysis for single and double exponential fitting for ATP induced dissociation of Acto·Myo19-3IQ by light scattering.** The residual analysis of the fitting to single (black scattered plot) or double (blue scattered plot) exponential functions of the transients obtained after mixing Acto·Myo19-3IQ (0.25 µM) with 7.8, 125 and 150 µM ATP (the experiments are fully described in Fig. 2 (main text).

Appendix Tables

Table S1

Constructs	K_{ATPase} (µM)	k_{cat} ($s^{-1}\cdot head^{-1}$)	*In vitro* motility ($nm\cdot s^{-1}$)	[Salt] mM	Light Chain	Protein determination method
***hs*Myo19-3IQ**	26.5 ± 5.4	2.7 ± 0.2	218 ± 39	50 (KCl)	Calmodulin	Not specified[1]
***mm*Myo19-3IQ**	20 ± 6.8	4.2 ± 0.72	46.8 ± 4.7	50 (NaCl)	LC9, LC12b	Coomassie Blue staining[2]

Table S1: Steady-state parameters and i*n vitro* motility velocities for Myo19-3IQ constructs according to Adikes *et al* [1] and Lu et al [2].

1 Adikes, R. C., Unrath, W. C., Yengo, C. M. & Quintero, O. A. Biochemical and bioinformatic analysis of the myosin-XIX motor domain. *Cytoskeleton* **70**, 281-295, doi:10.1002/cm.21110 (2013).

2 Lu, Z. *et al.* Mouse myosin-19 is a plus-end-directed, high-duty ratio molecular motor. *The Journal of biological chemistry* **289**, 18535-18548, doi:10.1074/jbc.M114.569087 (2014).

Table S2: Summary of Myo19-3IQ and Acto·Myo19-3IQ ATPase cycle rate and equilibrium constants

Parameter	***Value***	***Signal***
ATP binding		
k_{+1T} ($\mu M^{-1}s^{-1}$)	6.9 ± 0.2	mT
k_{-1T} (s-1)	0.3 ± 0.2	mT[1]
K_{1T} (μM)	0.04 ± 0.03	mT (k_{-1T}/k_{+1T})
k'_{+1T} ($\mu M^{-1}s^{-1}$)	4.4 ± 0.1	mT
k'_{-1T} (s^{-1})	1.0 ± 0.7	mT[1]
K'_{1T} (μM)	0.23 ± 0.16	mT (k'_{-1D}/k'_{+1D})[2]
ATP hydrolysis		
K_H	1.07 ± 0.08	Malachite green
$k_{+H} + k_{-H}$ (s^{-1})	129 ± 34	Malachite green
ADP binding		
k_{+1mD} ($\mu M^{-1}s^{-1}$)	31.8 ± 1.6	mD ($k_{obs,\ fast}$)
k_{-1mD} (s^{-1})	≈ 0	mD[1]
K_{1mD} (μM)	≈ 0	mD (k_{-1mD}/k_{+1mD})[2]
$^1/K_{mD,isom}$ (μM)	≈ 1	mD (A_{slow})
$k_{mD,isom}$ (s^{-1})	20 ± 7	mD ($k_{obs,\ slow,\ max}$)
k_{off} (s^{-1})	≈ 0	mD[1]
	10.8 ± 1.2	mD ($k_{obs,\ diss}$)
k_{+2mD} (s^{-1})	≈ 10.8	mD ($k_{off} = k_{-1mD} + k_{+2mD}$)
k_{-2mD} (s^{-1})	≈ 9.2	mD ($k_{mD,isom} = k_{+2mD} + k_{-2mD}$)
K_{2mD}	≈ 0.9	mD (k_{-2mD}/k_{+2mD})
$K_{D,overall}$ (μM)	<< 1	mD
k'_{+1mD} ($\mu M^{-1}s^{-1}$)	22.5 ± 0.9	mD ($k'_{obs,\ fast}$)[2]
k'_{-1mD} (s^{-1})	≈ 0	mD[1]
K'_{1mD} (μM)	≈ 0	mD (k'_{-1D}/k'_{+1D})[2]
$^1/K'_{mD,isom}$ (μM)	≈ 0.3	mD (A'_{slow})
$k'_{mD,isom}$ (s^{-1})	7.2 ± 1.2	mD ($k'_{slow,\ max}$)
k'_{off} (s^{-1})	≈ 0	mD[1]
	4.6 ± 0.4	mD ($k'_{obs,diss}$)
k'_{+2mD} (s^{-1})	≈ 4.6	mD ($k'_{off} = k'_{-1mD} + k'_{+2mD}$)
k'_{-2mD} (s^{-1})	≈ 2.6	mD ($k_{mD,isom} = k_{+2mD} + k_{-2mD}$)
K'_{2mD}	≈ 0.6	mD (k'_{-2mD}/k'_{+2mD})
$K'_{D,overall}$ (μM)	<< 1	mD[2]
P_i release		
k'_{-P_i} (s^{-1})	>172 ± 6	P_iBP[3]

[a]Conditions: 20 mM MOPS, pH 7.3, 50 mM KCl, 2 mM $MgCl_2$, 0.2 mM EGTA, 1 mM DTT, 25 °C.
[1]Calculated parameter from y-intercept of k_{obs} versus concentration of ligand
[2]Calculated parameter from rates or/and equilibrium constants
[3]Measured at 80 μM Actin

Table S3: DNA primers used to generate the plasmids used in this work

Primer	Construct Name	Oligos (5'- 3')
1	pF4A[1] Calmodulin-N	CGCGATCGCCATGGCTGATCAACTGACAGAAGAG
2	pF4A[1] Calmodulin-C	CGTTTAAACCTTCGCTGTCATCATCTGTACAAACTC
3	pFC14K[1] Myo19-3IQ[2]- N	CGCGATCGCCATGCTCCAGCAGGTCAATGGCC
4	pFC14K[1] Myo19-3IQ[2]- C	CGTTTAAACGCTCAGGGAACAGGGAGCTTGAG

[1]p4A and pFC14K are trademarks of Promega.
[2]Myo19-3IQ primers covers amino acids residues 1-848
